# Kaposi’s sarcoma of the conjunctiva and the eyelid leads to the diagnosis of human immunodeficiency virus infection – a case report

**DOI:** 10.1186/s12885-018-4611-3

**Published:** 2018-07-03

**Authors:** Filipe Sousa Neves, Joana Braga, João Cardoso da Costa, Joaquim Sequeira, Sandra Prazeres

**Affiliations:** Department of Ophthalmology, Centro Hospitalar Vila Nova de Gaia/Espinho, Avenida Cidade de Montgeron, 212 –, 4490-402 Póvoa de Varzim, Portugal

**Keywords:** HIV, Ocular, Conjunctiva, Eyelid, Kaposi sarcoma

## Abstract

**Background:**

The purpose of this case report is to describe a conjunctiva and eyelid Kaposi’s sarcoma (KS) as the initial manifestation of acquired immunodeficiency syndrome (AIDS), which led to the diagnosis of HIV infection. There are only 3 reported cases of ocular KS as an initial manifestation of HIV infection.

**Case presentation:**

A 32-year old white man presented to our department with a 1 month history of eye redness. The patient had an enlarged violet-coloured mass on the right superior eyelid which had evolved over the course of 1 week. There was also a mobile bulbar conjunctival lesion with a bright red colour, approximately 5 mm × 5 mm, in the superior temporal quadrant of his left eye. The lesions looked like a chalazion and a subconjunctival haemorrhage, respectivly. Presumed KS diagnosis was confirmed with HIV-1 positive testing and histopathology from tissue biopsy. The patient’s CD4 count was 23/mm^3^ and viral RNA load 427,000/ml. Further systemic evaluation showed a diffuse sarcoma.

**Conclusion:**

This case report demonstrates the importance of recognizing the ocular manifestations of AIDS in establishing the correct diagnosis of KS and subsequently diagnosing occult HIV infection. Although ocular KS as the initial manifestation of HIV-AIDS is an extremely rare event, a proper diagnosis may contribute to prompt management with personal and social relevance.

## Background

Kaposi’s sarcoma (KS) is the most common tumour in patients with the human immunodeficiency virus (HIV) infection and fully developed acquired immunodeficiency syndrome (AIDS). KS is a multifocal systemic disease related to the human herpes-virus 8 infection, found in patients with a low CD4 cell count: less than 500cell/μl, typically below 200. However, ocular involvement of this endothelial malignant tumour reported as AIDS-defining illness, is an exceptionally rare event [1, 2].

In the literature, there is no evidence-based algorithm for the treatment of ocular KS [3]. Nevertheless, there are reports of a successful management of eyelid and conjunctiva KS with a variety of therapies (alone or in conjunction), mainly dependent on location, size, number of lesions and extra-ocular involvement. Systemic approaches include highly active antiretroviral therapy (HAART) [3–6] and chemotherapy [4, 6]. Focal ocular lesions can be managed by surgical resection, radiation, cryotherapy or intralesional chemotherapy [7, 8]. Nowadays, HAART is compulsory to achieve systemic disease control in HIV-AIDS patients [3].

## Case presentation

**Fig. 1:**
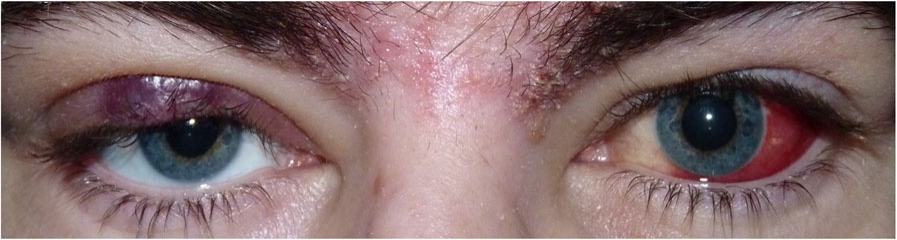
Kaposi’s sarcoma masquerading as a chalazion and a subconjunctival haemorrhage in the right upper eyelid and the left bulbar conjunctiva respectively

**Fig. 2:**
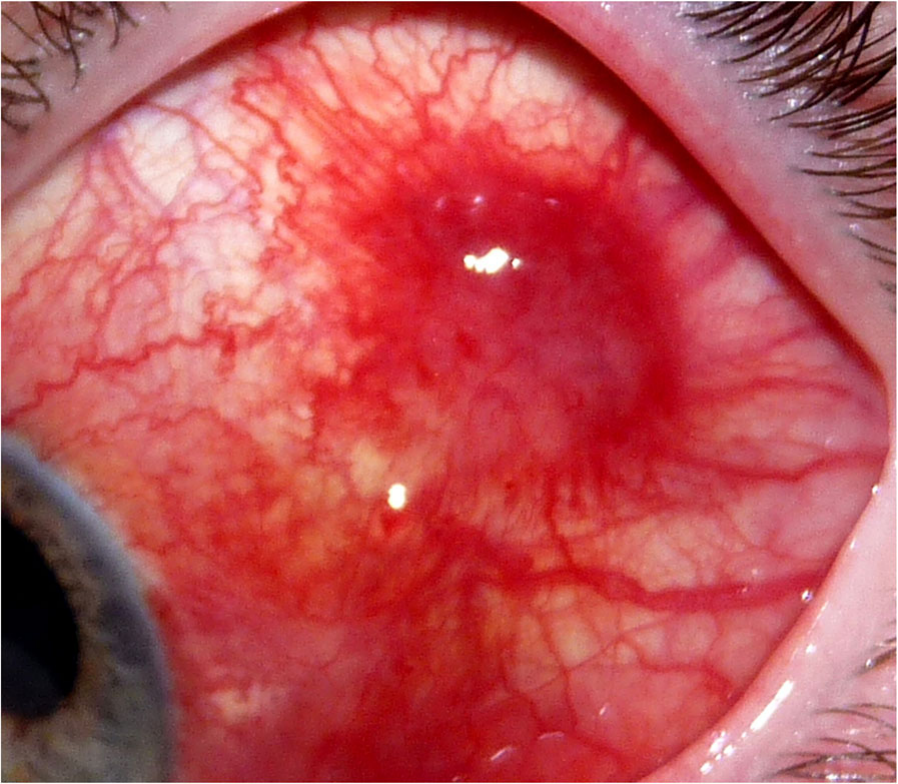
Kaposi’s sarcoma of the left superior temporal bulbar conjunctiva presenting as a painless red mass measuring approximately 5 mm × 5 mm

A 32-year old white man presented to our department with a 1 month history of eye redness (left eye). He had already been observed by a general practitioner who advised the patient to seek ophthalmological advice if the lesion would not resolve within 3 weeks. In the first ophthalmic evaluation, the patient presented with 2 ocular lesions. These were best observed in biomicroscopy. There was an enlarged violet-coloured mass on the right superior eyelid which had evolved over the course of 1 week (Fig. 1). There was also a mobile bulbar conjunctival lesion with a bright red colour, approximately 5 mm × 5 mm, in the superior temporal quadrant of his left eye (Figs. 1 and 2). The patient did not mention any pain or visual changes. The lesions looked like a chalazion and a subconjunctival haemorrhage, respectively (Fig. 1). Best corrected visual acuity was 20/20 in both eyes (Snellen chart) and subsequent fundoscopic exam was normal. Patient denied previous trauma history or drug abuse. Other systemic features included facial seborrheic dermatitis (Fig. 1), a characteristic of HIV-AIDS. Past medical history was unremarkable with no serological evaluation for HIV. The main differential diagnosis at the time of presentation was blood dyscrasia, due to the duplicity of lesions. We also considered KS as part of an immunodeficiency syndrome unknown to the patient. Blood cell count and differential analysis were normal and coagulation disorders were excluded. However the HIV-1 test was found positive and the presumed diagnosis of ocular KS was established. The patient elected for an excisional biopsy of the conjunctival mass and for an incisional biopsy of the right superior eyelid. In addition, HIV-AIDS staging was performed. A CD4 cell count was determined to be 23/mm^3^ and the viral RNA load of 427,000/ml. Pathologic examination confirmed KS diagnosis of both lesions. Postoperative evaluations were uneventful and no signs of recurrence were noticed during the 6-month follow-up.

Further multiple disciplinary assessments showed a diffuse KS with skin and supraglottis involvement. The patient was offered treatment for both AIDS and diffused KS. In order to achieve disease control, patient initiated systemic antiretroviral therapy – HAART - and systemic chemotherapy under medical supervision with regression of the tumour.

## Discussion and conclusions

Kaposi’s sarcoma is the most common neoplasm in AIDS patients [1, 2]. However, ocular involvement leading to the HIV infection diagnosis is exceptionally atypical. To our knowledge, there are only 3 reported cases of ocular KS as an initial manifestation of HIV infection. In all these reports, conjunctiva was the location of the tumour [9–11]. Nonetheless, there are also 4 cases of HIV-infected patients in which KS of the eye was the AIDS defining disease [12–15].

Fortunately, after HAART introduction in 1997, HIV-AIDS patients with KS are not seen as often in occidental societies [3]. This unique tumour can be similar to a subconjunctival haemorrhage. Therefore, physicians must be aware of ocular manifestations of AIDS, as one should not misdiagnose KS.

This case report shows the importance of accurately identifying AIDS ocular involvement. The knowledge about ocular lesions in AIDS led to the correct diagnosis of KS and subsequently the identification of occult HIV infection. Although ocular KS as the first clinical sign of HIV-AIDS is an extremely rare event, a prompt diagnosis can lead to a vital intervention in the patient’s own health and utmost social relevance.

## Abbreviations

AIDS: Acquired immunodeficiency syndrome
HAART: Highly active antiretroviral therapy
HIV: Human immunodeficiency virus
KS: Kaposi’s sarcoma
